# Concomitant rapidly growing aneurysm of intracavernous carotid artery and cavernous sinus thrombosis: Case report and review of the literature

**DOI:** 10.1097/MD.0000000000039022

**Published:** 2024-07-26

**Authors:** Yaoyao Shen, Fan Hu, Lingfeng Wu, Hongbing Nie

**Affiliations:** a Department of Neurology, Jiangxi Provincial People’s Hospital, The First Affiliated Hospital of Nanchang Medical College, Nanchang, Jiangxi Province, China; b Department of Neurology, Xiangya Hospital, Central South University, Jiangxi, National Regional Center for Neurological Diseases, Nanchang, Jiangxi Province, China.

**Keywords:** cavernous sinus thrombosis, endovascular treatment, intracavernous carotid artery, intracavernous infectious aneurysm, literature review

## Abstract

**Rationale:**

Intracavernous infectious aneurysm (ICIA), represents a rare entity that is always described in the form of case reports in the literature. The coexistence of ICIA and cavernous sinus thrombosis (CST) is extremely rare and poorly understood.

**Patient concerns:**

A 53-year-old female patient presented to our hospital with headache, nausea and fatigue for 3 weeks. She complained of blurry vision and drooping eyelids before admission. Neurological examination revealed bilateral decreased visual acuity, limitation of extraocular movements and decreased sensation of forehead. Brain magnetic resonance imaging (MRI) showed mixed signal intensities in both cavernous sinuses and expansion of right superior ophthalmic vein, suggesting the formation of CST. One month later, computed tomography angiography (CTA) confirmed a large aneurysm was attached to the left intracavernous carotid artery (ICCA).

**Diagnoese:**

This patient was diagnosed with ICIA and CST.

**Interventions:**

She was administered with intravenous meropenem and vancomycin and subcutaneous injection of low molecular heparin for 4 weeks.

**Outcomes:**

One month later, her extraocular movement had significantly improved, without ptosis and conjunctival congestion. At 1-year follow-up, her ophthalmoplegia fully recovered. Fortunately, such large aneurysm did not rupture in spite of slight broadening.

**Lessons:**

The coexistence of ICIA and CST is extremely rare. Contiguous infection from adjacent tissues is the foremost cause of ICIA. A repeated angiographic examination is recommended under enough anti-infective treatment due to the characteristics of rapid emergence and fast growth of infectious aneurysms.

## 1. Introduction

Intracranial infectious aneurysm (IIA), a rare neurological complication of systemic or central nervous infections, accounts for 0.5% to 6.5% of intracranial aneurysms.^[1,2]^ Pathogen infection leads to destruction of the arterial wall via hematogenous spread or direct extension of extravascular neighboring source.^[3,4]^ The vast majority of infectious aneurysms are associated with bacterial endocarditis, especially in patients with prosthetic valves, nosocomially acquired bloodstream infections, or a history of intravenous drug use.^[3]^ Compared with other intracranial aneurysms, IIA tends to occur in younger people and carries a higher mortality. It is estimated that the overall mortality of patients with IIA is 18.7% to 46.0%.^[2,5,6]^ Therefore, it is very crucial for clinicians to make early diagnosis and timely treatment in case of emergency. To our knowledge, IIA located on intracavernous carotid artery (ICCA) is relatively rare, with fewer than 50 cases reported in the literature so far.^[7]^ It is worth noting that some of these patients have intracavernous infectious aneurysms (ICIA) as well as cavernous sinus thrombosis (CST). As far as we know, CST is a rare, severe, thrombophlebitic process and typically arises from infection of the paranasal sinuses.^[8]^ Septic CST is one of the main causes of ICIA. Here, we describe a rare case of concomitant CST and ICIA, and present a literature review.

## 2. Case presentation

**Figure 1.**
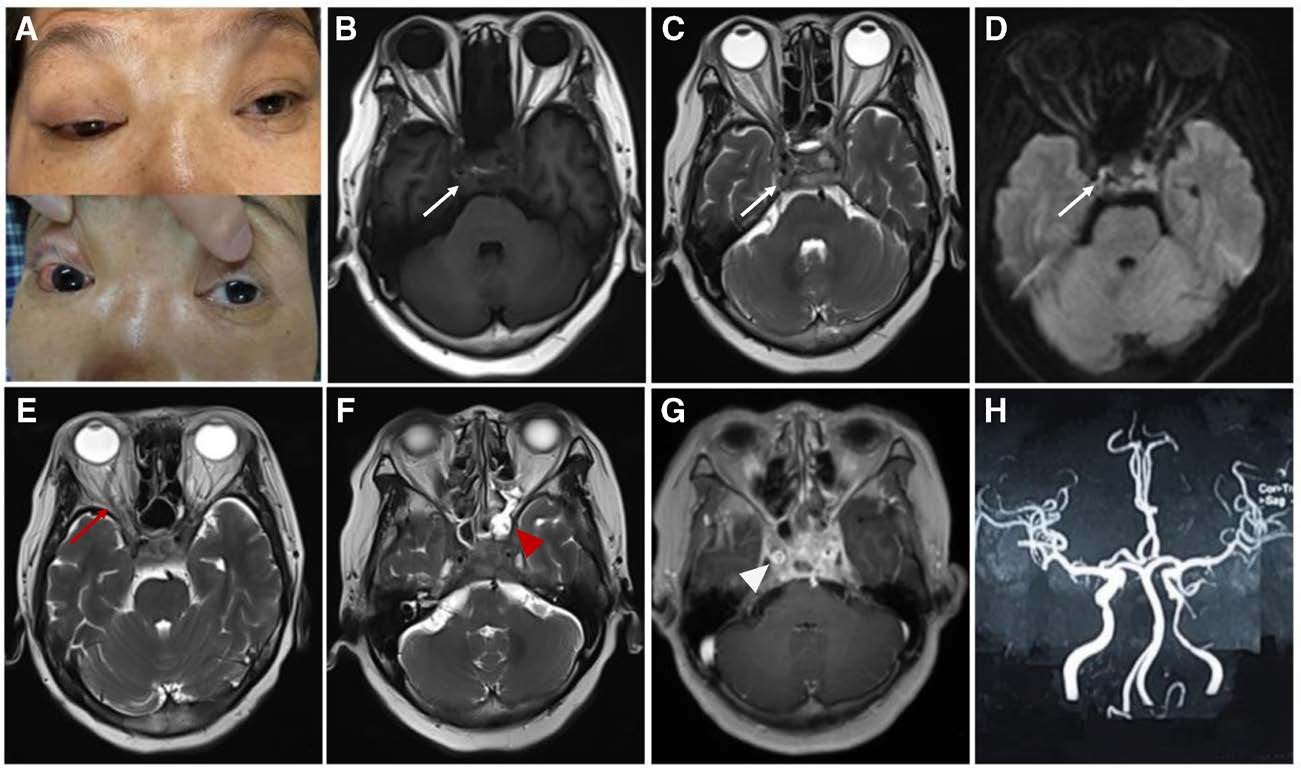
On admission, neurological examination revealed bilateral ptosis and right exophthalmos and conjunctival congestion (A). On day 3 after admission, brain MRI showed mixed signal intensities in both cavernous sinuses on T1-weighted, T2-weighted and diffusion-weighted images (white arrows) (B-D). Axial T2-weighted images revealed expansion of right superior ophthalmic vein (red arrow) and sphenoid and ethmoid sinusitis (red arrowhead) (E, F). Axial Gadolinium enhanced-T1-weighted image demonstrated expansion of both cavernous sinuses with circular enhancement of the arterial walls (white arrowhead) (G). MRA disclosed stenosis of the left ICCA (H). ICCA = intracavernous carotid artery, MRA = magnetic resonance angiography, MRI = magnetic resonance imaging.

A previously healthy 53-year-old woman was admitted to our hospital owing to a 3-week history of headache, nausea and fatigue. The headache was paroxysmal pricking pain and mainly located at forehead. 3 days prior to admission, she complained of left ptosis and blurry vision. Two days later, the right eyelid also drooped, with homolateral exophthalmos and conjunctival congestion. On admission, the patient was alert and fully oriented to time and place. Her vital signs were stable with temperature of 36.9°C, heart rate of 59 beats per minute, respiratory rate of 18 breaths per minute, and blood pressure of 115/68 mm Hg. Neurological examination revealed bilateral visual impairment (0.3 in both eyes). There was an obvious limitation of extraocular movements in all directions (like frozen eye) as well as ptosis, bilaterally (Fig. 1A). Both pupils were equal, round and sensitive to light. Facial sensory examination showed reduction to pinprick over the forehead in a V1 distribution, but corneal reflexes were reserved bilaterally. Other neurological abnormalities, including sensory and motor deficits of limbs, Kernig sign, and Babinski sign, were absent. Laboratory investigations showed a white cell count of 11.5 × 10^9^/L (reference range 3.5–9.5 × 10^9^/L) with 88.3% neutrophils, hemoglobin of 104 g/L (reference range 110–150 g/L), C-reactive protein of 137 mg/L (reference range 0–10 mg/L), D-dimer of 0.55 mg/L (reference range 0–0.24 mg/L), fibrinogen of 6.22 g/L (reference range 2–4 g/L), glycosylated hemoglobin of 6.1% (reference range 4.0–6.0%), and potassium of 3.3 mmol/L (reference range 3.5–5.3 mmol/L). Serum concentration of triiodothyronine (T3) and free T3 were 0.47 ng/mL (reference range 0.8–2.0 ng/mL) and 2.92 pmol/L (reference range 3.1–6.8 pmol/L), respectively. Test of antinuclear antibody spectrum screening of serum was negative except for anticardiolipin IgM with 29.9 MPU (normal < 10 MPU).

On day 2 after admission, chest computed tomography (CT) demonstrated infection of the lower lobes of both lungs. Cardiac and abdominal doppler ultrasound were unremarkable. The diagnosis of cavernous sinus syndrome was quickly established based on multiple cranial nerve (CN) palsy (III-VI), decreased visual acuity, proptosis and conjunctival congestion. The next day, brain magnetic resonance imaging (MRI) was subsequently performed and depicted mixed signal intensities in both cavernous sinuses on T1-weighted and T2-weighted images with partly restricted diffusion (Fig. 1B–D). Besides, there was an expansion of right superior ophthalmic vein as well as sphenoid and ethmoid sinusitis on T2-weighted images (Fig. 1E and F). Contrast-enhanced T1-weighted image illustrated both cavernous sinuses were enlarged with circular enhancement of the arterial walls (Fig. 1G). Stenosis of the left cavernous carotid artery was distinctly visualized on magnetic resonance angiography (MRA) (Fig. 1H). On the same day, cerebrospinal fluid (CSF) analysis yielded slightly increased opening pressure (200 mmH_2_O) and cell count (20 cells/mm^3^), with normal protein, glucose, and chloride. Microbiological investigations, including bacterial culture, virus polymerase chain reaction, India Ink preparation, T-spot, and acid-fast bacilli smear, were all unremarkable. In addition, consecutive cultures of blood and sputum demonstrated negative results.

**Figure 2.**
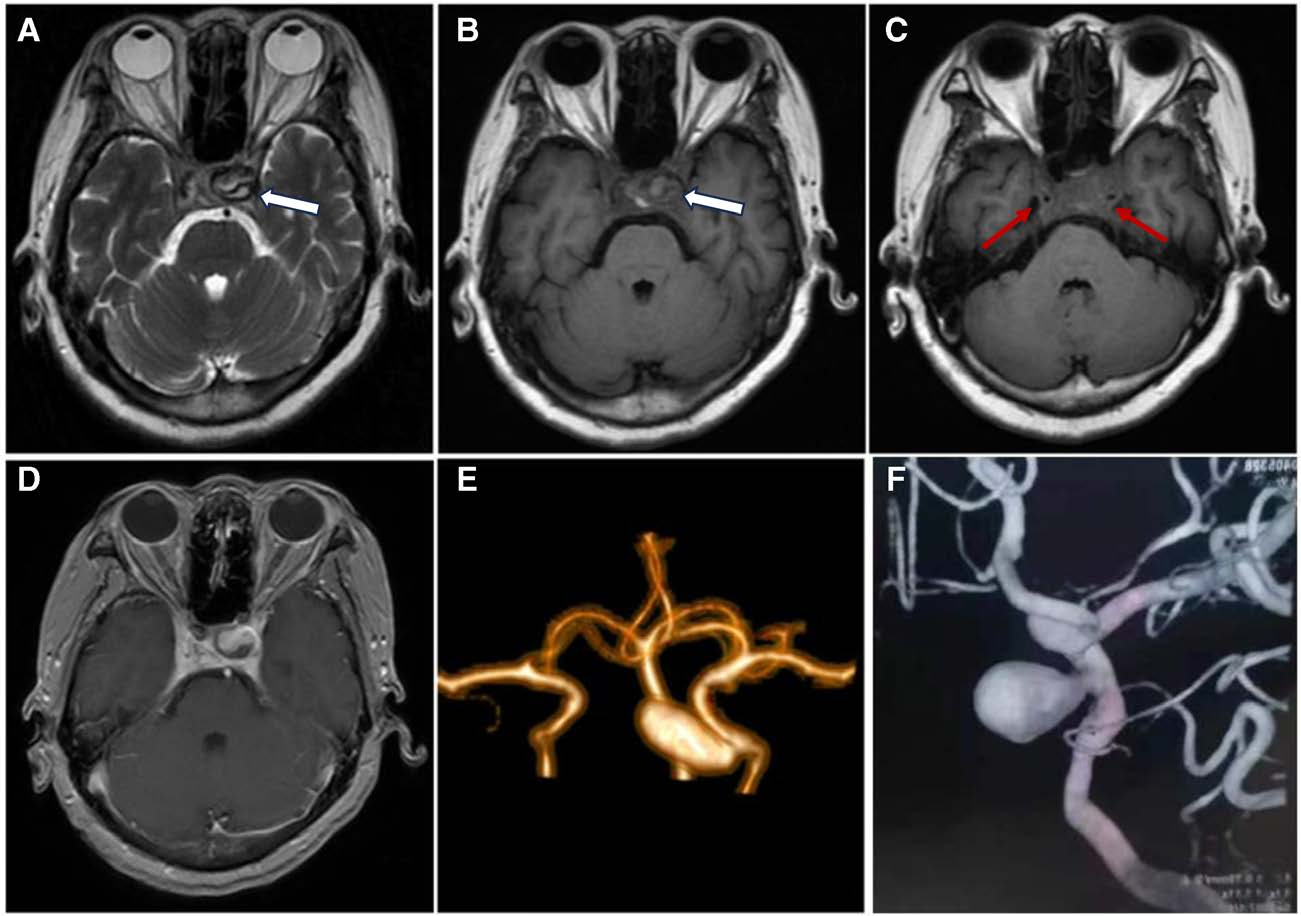
On day 30 after admission, axial T1-weighted and T2-weighted images showed twisty walls of left ICCA (white arrows) and narrow lumens of both ICCAs (red arrows) (A-C). Contrast-enhanced MRI showed the size of bilateral cavernous sinuses shrank (D). CTA confirmed a fusiform aneurysm was located on the left ICCA (E). At 1-year follow-up, left internal carotid angiography demonstrated a slightly widened aneurysm (F). CTA = computed tomography angiography, ICCA = intracavernous carotid artery, MRI = magnetic resonance imaging.

The clinical presentations together with typical neuroradiological findings fulfilled the diagnosis of CST. Whereafter, a neurosurgery consultation was quickly requested and anti-infective treatment was primarily recommended. On day 4 after admission, she was administered with intravenous meropenem and vancomycin and subcutaneous injection of low molecular heparin (4000IU, twice a day) for 4 weeks. At the end of treatment, she had a visual acuity of 0.5 bilaterally. Moreover, extraocular movement in all directions had significantly improved, without ptosis and conjunctival congestion. However, there was still insufficient movement of right gaze. The pupil on the left is larger than that on the right (left 4.5 mm and right 3 mm), and the former is insensitive to light. A repeated MRI on day 30 after admission showed twisty walls of left ICCA and narrow lumens of both ICCAs (Fig. 2A–C). Contrast-enhanced MRI revealed the size of bilateral cavernous sinuses shrank (Fig. 2D). Computed tomography angiography (CTA) confirmed a large aneurysm was unexpectedly attached to the left ICCA (Fig. 2E). In consideration of high risk of operation, the patient and her family members finally refused embolization of intracranial aneurysm. Antiplatelet therapy with aspirin (100mg per day) was subsequently started following low molecular heparin in order to decrease the risk of ischemic stroke and she was discharged on day 34 after admission. At 1-year follow-up, her ophthalmoplegia fully recovered. Fortunately, such large aneurysm did not rupture in spite of slight broadening (Fig. 2F).

Furthermore, a literature search for reported cases of such co-occurrence in the English language publications between 1960 and August 2023 was performed using Web of Science, PubMed, and Embase databases. A total of 22 patients with concurrent ICIA and CST, including our case, were incorporated in this literature review. The clinical data of included patients was summarized in Table 1. Among these patients, 14 (64%) were men. The age at onset ranged from 3 to 76 years, and the mean age was 31.1 years. The vast majority of potentially infectious sources of patients with co-occurrence of ICIA and CST resulted from adjacent organs, including paranasal sinuses, nose, orbit, eye, tooth, and face, but only 1 originated from infective endocarditis. In this review, fourteen of 22 patients (64%) had positive culture results, in which the majority were blood samples (n = 9). Only 1 case had positive results from CSF culture. In positive culture results, *Staphylococcus aureus* (n = 5) was the most common pathogen. Other identified organisms included fungus (n = 3), *Streptococcus sp.* (n = 2), *Fusobacterium* (n = 2), *Propionibacterium* (n = 1). Besides, Gram-negative bacillin has been reported in 1 case, but without further identification. Regarding neurological signs during hospitalization, ophthalmoplegia was presented in 22 patients (100%), exophthalmos in 11 (50%), chemosis in 7 (32%), ptosis in 8 (36%), decreased visual acuity in 10 (45%), neck stiffness in 2 (9%), hemiparesis in 2 (9%), facial palsy in 1 (5%), 5th CN palsy in 3 (14%). Total ophthalmoplegia accounted for more than half of all individuals. Aneurysm of ICCA was found in all patients on angiographic examination and 2 of these patients had concomitant aneurysms in other places. In addition, ICIA complicated with parent artery stenosis was visualized in 4 patients (18%).

**Table 1:** Cases of co-occurrence of ICIA and CST in the literature.

Case	Age/sex	Potential source	Pathogen	Neurological sign	Angiographic finding	Treatment	Aneurysm outcome	Clinical outcome
1^[9]^	6/M	Eye infection	*S aureus* (blood)	Bilateral total ophthalmoplegia, exophthalmos and chemosis, dilated and fixed pupil on the right eye	Aneurysm of left ICCA, occlusion of right ICA,	Medication	Reduction in size	PR
2^[9]^	6/F	Eye infection	*S aureus* (blood)	Total ophthalmoplegia, exophthalmos, chemosis, and ptosis on the right eye	Aneurysm of right ICCA	Medication	Complete regression	CR
3^[9]^	10/M	Eye infection	*S aureus* (blood an CSF)	Somnolence, left hemiparesis, total ophthalmoplegia and exophthalmos of the right eye, neck stiffness	Aneurysm of right ICCA, small aneurysms at the right MCA and pericallosal artery	Medication	Loss to follow-up	Loss to follow-up
4^[10]^	59/M	Uncertain	ND	Total ophthalmoplegia on the right eye	Aneurysm of right ICCA	Medication, ligation of CCA	No	PR
5^[11]^	19/M	Facial cellulitis	*S aureus* (blood)	Peripheral facial palsy, bilateral total ophthalmoplegia, exophthalmos and ptosis	Aneurysm of left ICCA	Medication, balloon occlusion	No filling	CR
6^[12]^	50/F	Orbital cellulitis	Negative	Right exophthalmos and chemosis, right VI CN palsy	Aneurysm of left ICCA	Medication	No	Death
7^[13]^	48/F	Tooth abscess	*Streptococcus sp.* (CSF)	Right III and VI CN palsies, exophthalmos	Aneurysm of right ICCA	Medication, balloon occlusion	Reduction in size	CR
8^[14]^	63/M	Sinusitis	Negative	Left total ophthalmoplegia, decreased visual acuity in right eye	Aneurysm of left ICCA	Medication, ligation of ICA	No	PR
9^[15]^	19/M	Sinusitis	Negative	Left visual loss and VI CN palsy	Aneurysm of left ICCA, giant ophthalmic aneurysm	Medication, coil occlusion	No filling	PR
10^[16]^	23/M	Sinusitis	*Propionibacterium sp.* (blood)	Decreased visual acuity, exophthalmos and chemosis in the right eye, right III, IV, V, and VI CN palsies, left hemiparesis	Aneurysm of right ICCA	Medication, balloon occlusion	No filling	PR
11^[17]^	46/M	Subdural empyema	*Streptococcus con*stell*atus* (blood)	Bilateral total ophthalmoplegia and ptosis, neck stiffness	Aneurysm of bilateral ICCAs with parent arteries stenosis	Medication, drainage, coronary stent, coil occlusion	No filling	PR
12^[18]^	76/M	Sinusitis	*Aspergillus sp.* (histopathology)	Exophthalmos and ophthalmoplegia on the right eye	Aneurysm of right ICCA	Medication, coil occlusion	No filling	Death (CRF)
13^[19]^	22/M	Infective endocarditis	*Candida albicans* (blood)	Binocular horizontal diplopia, abduction deficit and ptosis of the left eye	Aneurysm of left ICCA	Medication, valve replacement	Loss to follow-up	CR
14^[20]^	10/M	Meningitis	ND	Decreased visual acuity, left ophthalmoplegia with ptosis	Aneurysm of left ICCA	Medication, coil occlusion	No filling	CR
15^[20]^	3/M	Orbital cellulitis	ND	Decreased visual acuity, abduction deficit of the left eye	Aneurysm of both ICCAs	Medication	No	NI
16^[21]^	32/M	Periorbital abscess	*Gram-negative bacilli* (abscess)	Bilateral limited eye movement, left ptosis	Aneurysm of left ICCA, stenosis of bilateral ICCAs	Medication, STA-MCA anastomosis, ligation of ICA	No	CR
17^[22]^	11/F	Nosal infection	Negative	Total ophthalmoplegia, reduced visual acuity and ptosis of the right eye, bilateral chemosis	Narrowing of right ICCA with aneurysm	Medication, covered stent placement	No filling	CR
18^[23]^	62/F	Uncertain	*Fusobacterium* (blood)	Total ophthalmoplegia, reduced visual acuity, and exophthalmos of the right eye	Aneurysm of right ICCA	Medication, coil occlusion	No filling	CR
19^[24]^	41/F	Suprasellar cellulitis	*Aspergillus* *fumigatus* (sinus)	Loss of vision of the left eye, bilateral III and left V CN palsies	Aneurysm of right ICCA, narrowing of right ICA	Medication, flow-diverting stents	No filling	PR
20^[25]^	7/F	Facial infection	*S aureus* (pus)	Ophthalmoplegia, chemosis, exophthalmos and on the left eye, right ptosis	Aneurysm of right ICCA	Medication	No	PR
21^[26]^	18/M	Sinusitis	*F necrophorum* (blood)	Left total ophthalmoplegia and reduced visual acuity, loss of left V1 CN sensation	Aneurysm of left ICCA	Medication, coil occlusion	No	CR
22 (present case)	53/F	Sinusitis	Negative	Bilateral total ophthalmoplegia and reduced visual acuity, chemosis and exophthalmos of the right eye	Aneurysm of left ICCA	Medication	No change	CR

CCA = common carotid artery, CN = cranial nerve, CR = complete recovery, CRF = cardiorespiratory failure, CSF = cerebrospinal fluid, F = female, ICA = internal carotid artery, ICCA = intracavernous carotid artery, ICIA = intracavernous infectious aneurysm, M = male, MCA = middle cerebral artery, ND = not described, NI = no improvement, PR = partial recovery, STA-MCA = superficial temporal-middle cerebral artery.

In the literature, the treatment approaches of ICIA were diverse. Generally, all included patients were administrated with prolonged intravenous antibiotics, initially empiric with broad-spectrum and then specific in accordance with culture and sensitivities. Fourteen of 22 patients received operative treatment for ICIA, including coil occlusion (n = 6), ligation of CCA or ICA (n = 3), balloon occlusion (n = 3), and stent placement (n = 2). Among 11 patients with endovascular treatment, almost all patients achieved well angiographic outcomes except for 1 lack of detailed description of surgical procedure. In the remaining 8 patients with conservative treatment, complete regression was seen in only 1 patient, reduction in 1, no change in 1, loss to follow-up in 2, and no record in 3. Regarding clinical outcome, complete recovery was presented in 10 patients, partial recovery in 8, no improvement in 1, loss to follow-up in 1, and death in 2. Case 6 and case 12 died of ruptured aneurysm and cardiorespiratory failure, respectively. The clinical remission rate in patients with operative treatment was up to 93% (13/14).

## 3. Discussion

Since Osler first description of mycotic endocarditis producing multiple aneurysms in 1885, the term “mycotic aneurysm” has been mistakenly used to describe any aneurysm that is secondary to infectious process. Nowadays, “bacterial aneurysm” and “mycotic aneurysm” are reserved for aneurysms derived from bacterial infection and fungal infection, respectively.^[10]^ IIA, a rare infectious vascular disease of the central nervous system, represents around 0.5% to 6.5% of all intracranial aneurysms.^[2,27–29]^ The etiologies of IIA can be divided into following 3 forms: septic embolization, such as such as infective endocarditis; contiguous infection, such as cavernous sinusitis; primary or cryptogenic aneurysm.^[2]^ The commonest etiology of IIA is septic embolization following acute or subacute infective endocarditis.^[30]^ ICIA is defined as IIA located on ICCA, representing a rare entity that is always documented in the form of case reports in the literature.^[22–26]^ Based on our review, the co-occurrence of ICIA and CST is more prevalent in men (64%). There is a wide age span of included patients, from children to old people, and the majority are young to middle-aged adults. The potential infectious sources largely stem from neighboring tissues, such as paranasal sinuses, nose, orbit, eye, tooth, or face, rather than from infective endocarditis. In those patients with positive culture results, identified organisms include bacteria and fungi, most commonly *S aureus*. However, viral and parasitic infections are absent in our review.

The pathogenesis of ICIA is not fully understood. As for septic embolization, friable microbial vegetated on diseased prosthetic or biological valves fall into arterial circulation and attaches to vessel wall of cerebral arteries. Subsequently, pathogens escape through vasa vasorum or the Virchow-Robin spaces of the vessel wall. This kind of mobile process of pathogens can lead to inflammatory infiltration of the outer membrane, and then of the medial and inner membranes.^[31,32]^ Release of inflammatory mediators further contributes to degradation of arterial wall and formation of aneurysm.^[33]^ Moreover, hydrostatic pulsation thrusts against the affected vessel wall resulting in aneurysmal growth.^[34]^ Some specialists have proposed an alternative hypothesis that septic emboli may block the vascular lumen and then spread from the intima to the adventitia, eventually leading to a similar pattern of vessel wall degradation.^[2,35,36]^

It should be emphasized that ICIA can also be caused by direct extension of extravascular infectious focus, such as septic CST.^[9,13,28]^ In this review, all included patients had CST, which developed as a result of the local spread of infection from an original source to the cavernous sinuses. Therefore, contiguous infection is the foremost cause of ICIA. To our knowledge, cavernous sinus, a venous plexus situated in the floor of the middle cranial fossa on either side of the sella turcica, is bordered by the anterior clinoid process and superior orbital fissure anteriorly and by the petrous temporal bone posteriorly.^[37]^ Cavernous sinus complicatedly communicates with head and neck venous system, with inflow from the sphenoparietal sinus and superior and inferior ophthalmic veins anteriorly, and outflow to the superior and inferior petrosal sinuses posteriorly and inferiorly.^[38]^ As intracranial venous system is lack of valves, bloods flowing into cavernous sinus could be either forward or retrograde. Those above-mentioned theories make cavernous sinus vulnerable to septic thrombosis that is originated from neighboring infection at multiple sites, such as orbits, midface, neck, or deep facial spaces. Furthermore, infection can also spread from adjacent tissues into cavernous sinus via breakdown or defects in the soft tissue or bone.^[39]^ The connection between cavernous sinus and sphenoid sinus is tight, and they are separated by thin bone or sometimes only soft tissue if the bone is not fully formed. Such anatomic feature makes it possible to spread infection between these 2 cavities. Just as our case presented here, she had no fever or predisposing heart disease. In addition, repeated transthoracic echocardiography and consecutive blood culture revealed normal findings. Hence, our patient did not fulfill the diagnostic criteria of infective endocarditis. We, therefore, speculated that infection disseminates from sphenoid sinus into cavernous sinus via the venous flow or direct destruction of the connected bone, and further invasion of inflammatory cells into the arterial wall eventually results in the formation of ICIA.

Up to now, few studies have illustrated the pathological features of ICIA. Sugie et al^[40]^ performed an autopsy study of ICIA, in which histological examination showed discontinuous vessel wall of the infected ICA, and inflammatory infiltration in the adventitia and adjacent tissues. Moreover, another autopsy study showed obvious neutrophil infiltration of the adventitia and media with reactive intimal proliferation, suggesting an associated subacute/chronic inflammatory focal angiitis that may weaken the vessel wall and promote aneurysmal formation.^[41]^ Clinical presentation of patients with concurrent ICIA and CST is based on local infection, aneurysmal compression, and obstruction of cavernous venous drainage. Headache and fever are the most common symptoms. Increased pressure in the cavernous sinus may cause multiple CNs paralysis, including ophthalmoplegia (palsies of III, IV and/or VI), ptosis, and facial hypesthesia in the distribution of V1 and/or V2. Hampering ophthalmic vein drainage may lead to exophthalmos, chemosis, eye pain, headache, and loss of vision. Simultaneously, funduscopic examination may disclose papilledema and/or retinal vein dilation. Compression of the plexus of post-ganglionic sympathetic neurons surrounding the ICA may generate a Horner syndrome. If there is accidental rupture of an ICIA, patient will present with carotid-cavernous fistula with symptoms and signs including exophthalmos, chemosis, ocular pain, visual loss, and conjunctival arterialization.^[8,42]^ In this review, the commonest neurological sign is ophthalmoplegia, which can be total or partial, followed by exophthalmos and loss of vision. Alteration of ocular signs either indicate progressive process of cavernous sinus syndrome or suggest formation of ICCA. Under the circumstances, a repeated angiographic examination should be conducted timely.

Presently, the diagnosis of ICIA is based on the verification of an aneurysm located on ICCA by neuroimaging examination as well as the presence of predisposing infectious conditions, such as -infective endocarditis, CST, meningitis, etc. The utilization of noninvasive angiographic techniques, such as MRA and CTA, is becoming more common practice in initial test for intracranial aneurysms. However, digital subtraction angiography remains the golden standard for testing IIA, especially small (i.e., <3 mm) or distal aneurysm. IIA is always rendered as fusiform or irregular, and may change in size and shape during follow-up.^[32]^ It is worth noting that IIA may rapidly emerge, fast grow or unexpectedly rupture within a short time. Hence, repeated angiographic examination for tracing alteration of aneurysm is very crucial during hospitalization. Currently, there are no guidelines for the management of ICIA. Treatment options for patients with ICIA include conservative medical management with antibiotic therapy, open cranial surgery, and endovascular intervention. Anti-infective treatment is obligatory and usually persists for 4 to 6 weeks. If ICIA does not subside or even enlarge after anti-infective therapy alone, a surgical clipping or endovascular therapy is required. In contrast with noninfectious aneurysms, infectious aneurysms tend to be more friable, making clipping difficult with high risk of rupture. Over the past 5 decades, the main surgical option for ICIA is endovascular therapy, including balloon occlusion, coil occlusion, endovascular stenting. With the advance of interventional therapy materials, endovascular therapy has gradually become the preferred method for patients with ICIA. Actually, rupture of ICIA is rare in this review. Venous pressure in the cavernous sinus plays an important role in restricting aneurysm growth. But more importantly, endovascular intervention prevents the aneurysm from rupture.

## 4. Conclusion

In summary, ICIA is a rare infectious vascular disease of the central nervous system. The main pathogenesis of the co-occurrence of ICIA and CST is direct extension of extravascular infectious focus. A timely angiographic examination is recommended due to the features of rapid emergence and fast growth of ICIA. As for ICIA, persistent anti-infective treatment is fundamental and even obligatory. If there is an alteration in size or shape of aneurysm under continuous neuroimaging monitoring, endovascular therapy should be taken into consideration.

## Acknowledgments

We thank the patient and her family for their cooperation. The authors also thank the Jiangxi Province Key Laboratory of Neurology (Grant No. 2024SSY06081) for its support in this work.

## Author contributions

**Conceptualization:** Yaoyao Shen, Lingfeng Wu.

**Data curation:** Yaoyao Shen, Fan Hu.

**Formal analysis:** Yaoyao Shen, Fan Hu.

**Supervision:** Hongbing Nie.

**Writing – original draft:** Yaoyao Shen.

**Writing – review & editing:** Lingfeng Wu, Hongbing Nie.
